# Enhanced Production of Bioactive Isoprenoid Compounds from Cell Suspension Cultures of *Artemisia annua* L. Using β-Cyclodextrins

**DOI:** 10.3390/ijms151019092

**Published:** 2014-10-21

**Authors:** Francesca Rizzello, Angelo De Paolis, Miriana Durante, Federica Blando, Giovanni Mita, Sofia Caretto

**Affiliations:** Istituto di Scienze delle Produzioni Alimentari—CNR, Via Prov.le Monteroni, 73100 Lecce, Italy; E-Mails: RizzelloFrancesca@libero.it (F.R.); angelo.depaolis@ispa.cnr.it (A.D.P.); miriana.durante@ispa.cnr.it (M.D.); federica.blando@ispa.cnr.it (F.B.); giovanni.mita@ispa.cnr.it (G.M.)

**Keywords:** plant cell cultures, cyclodextrins, *Artemisia annua* L., carotenoids, quinones

## Abstract

Plant cell cultures as valuable tools for the production of specific metabolites can be greatly improved by the application of elicitors including cyclodextrins (CDs) for enhancing the yields of the desired plant compounds. Here the effects of 2,6-dimethyl-β-cyclodextrins (DIMEB) on the production of carotenoids and quinones from *Artemisia annua* L. cell suspension cultures were investigated. The addition of 50 mM DIMEB induced an early increase of intracellular carotenoid and quinone contents, which could be observed to a higher extent for lutein (10-fold), Q9 (3-fold) and Q10 (2.5-fold). Real Time PCR analysis revealed that the expression of 1-deoxy-d-xylulose-5-phosphate reductoisomerase (*DXR*) gene in DIMEB treated cell cultures after three days was 2.5-fold higher than in untreated samples, thus suggesting that the DIMEB induced increase of carotenoids and quinones could be due to the induction of the plastidial isoprenoid biosynthetic route. In addition, the DIMEB treatment induced an enhanced release of carotenoids and quinones into the culture medium of *A. annua* cell suspension cultures possibly due to the ability of CDs to form inclusion complexes with hydrophobic molecules.

## 1. Introduction

Plant cell cultures have received great attention as a valuable tool for the production of specific metabolites. In recent years many efforts have been made in order to develop and optimize strategies for enhancing the yields of the desired plant metabolites by eliciting their biosynthesis or increasing the efficiency of product recovery. To this regard, release of secondary metabolites into the culture medium can be particularly helpful since it facilitates downstream production processing also at a commercial level [1].

Cyclodextrins are non-reducing cyclic oligomers of 1,4-α-d-linked glucopyranose units possessing a cone shape with a lipophilic cavity and a hydrophilic exterior. Beta-cyclodextrins (β-CDs), which are formed by seven β-d-glucopyranose units, can form inclusion complexes with guest molecules of a wide range of different molecular weights [2]. For this reason, they are largely used as complexing agents to increase the water solubility of various compounds [3]. Recently, β-CDs have been also used as agents able to induce secondary metabolism thus acting as elicitors in plant cell cultures by enhancing metabolite biosynthesis [4]. The ability of β-CDs to elicit resveratrol production in grapevine cell cultures was likely due to their chemical similarity to pectic oligosaccharides released from the cell wall after fungal infection [5]. Furthermore, when applied as elicitors, β-CDs enhanced the accumulation of specific metabolites outside the cells in the culture medium [6]. Thus, among the approaches to enhance secondary metabolite yields in plant cell cultures, the use of cyclodextrins has the potential to be biotechnologically relevant.

Terpenoids are a large family of natural products serving essential plant processes such as respiration (ubiquinone Q9 and Q10) and photosynthesis (carotenoids and chlorophylls) but also being important healthful compounds for humans due to the known powerful antioxidant activity [7,8]. Particularly, dietary carotenoids including β-carotene in the class of carotenes, and lutein and zeaxanthin in the class of xanthophylls, are considered to be beneficial in the prevention of a variety of major diseases, including certain cancers and eye diseases [9]. In plant cells, terpenoids can derive from two distinct biosynthetic routes, the cytosolic mevalonate (MVA) and the plastidial MVA-independent pathway. Although these routes operate independently in plants, there is a communication and an exchange of intermediates between the cytosolic and the plastidial pathways of terpenoid biosynthesis [10].

*Artemisia annua* L. represents one of the most known examples of plant used in traditional medicine as well as in modern pharmacology. The genus *Artemisia* is one of the largest of the family *Asteraceae* showing rich phytochemical diversity. For many centuries *A. annua* plant extracts were used against fever. The discovered antimalarial properties of this plant led to the identification of the compound artemisinin, a sesquiterpene lactone, active against malarial agents. Due to the importance of such metabolite, we established and characterized *A. annua* L. cell cultures as for the production of artemisinin and the response to different elicitors, such as methyl jasmonate and miconazole [11]. We also applied β-CDs which significantly increased the production of artemisinin and its release into the culture medium [6].

The aim of this work was to use these established *A. annua* L. suspension cultures to investigate the effects of β-CDs on the production of other important bioactive compounds belonging to the isoprenoid class such as carotenoids and quinones.

## 2. Results and Discussion

### 2.1. Effects of DIMEB on Intracellular and Extracellular Carotenoids and Quinones

In a previous study, chemically modified β-CDs (2,6-dimethyl-β-cyclodextrins, DIMEB) were effectively used to significantly increase the production and release into the culture medium of artemisinin in *Artemisia annua* suspension cell cultures [6]. To study the effects of DIMEB on the production of other important isoprenoid metabolites, carotenoids and quinones were analyzed in actively growing *A. annua* suspension cell cultures treated with DIMEB. The concentration of 50 mM for two different time intervals, three and seven days, was chosen on the basis of previous results obtained using different concentrations of DIMEB and various time intervals as for the production of the terpenoid artemisinin and according to recent literature data [6,12,13]. The effects of DIMEB on *A. annua* cell growth were previously reported showing that DIMEB had no negative effects on the growth of these cultures [6]. The viability assay, carried out using the fluorescein diacetate staining method [14], confirmed that DIMEB treatments did not affect the viability of the cultures (data not shown). This indicates that the addition of CDs to the culture medium does not affect the growth ability of plant cell cultures as also reported by other authors in *Vitis vinifera* cell cultures [15].

**Figure 1:**
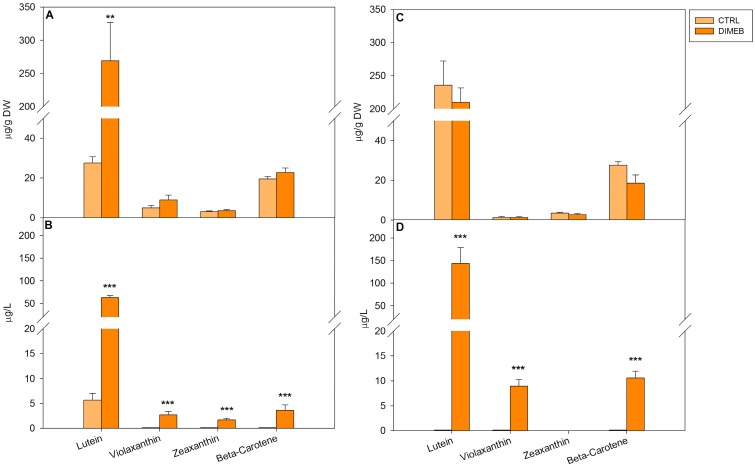
Intracellular and extracellular carotenoid levels in *A. annua* cell cultures untreated (CTRL) or treated (DIMEB) with 50 mM DIMEB. Data are expressed as mean ± standard deviation from three independent experiments, ** *p* < 0.01; *** *p* < 0.001 in comparison with control assessed by Anova-one-way *post hoc* Holm-Sidak test. Intracellular (**A**) and extracellular (**B**) carotenoid levels after three days of treatment; intracellular (**C**) and extracellular (**D**) carotenoid levels after seven days of treatment.

**Figure 2:**
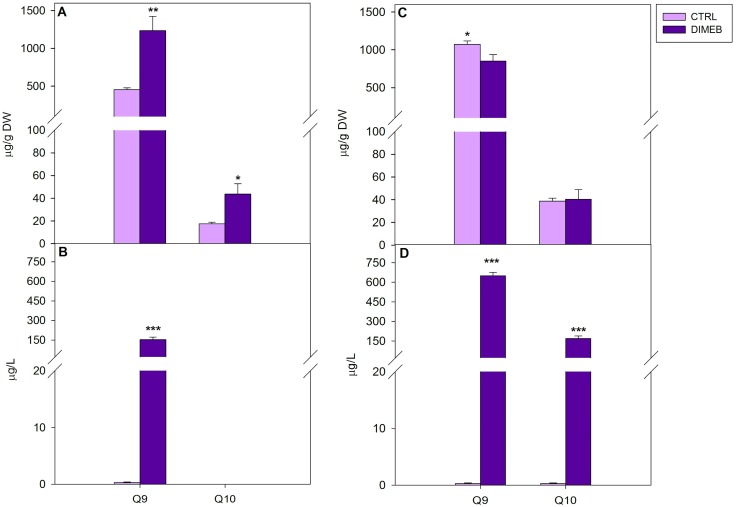
Intracellular and extracellular quinone levels in *A. annua* cell cultures untreated (CTRL) or treated (DIMEB) with 50 mM DIMEB. Data are expressed as mean ± standard deviation from three independent experiments, * *p* < 0.05; ** *p* < 0.01; *** *p* < 0.001 in comparison with control assessed by Anova-one-way *post hoc* Holm-Sidak test. Intracellular (**A**) and extracellular (**B**) quinone levels after three days of treatment; intracellular (**C**) and extracellular (**D**) quinone levels after seven days of treatment.

When carotenoids were analyzed in cell extracts of *A. annua* suspension cultures, cells treated for three days with 50 mM DIMEB showed slight increases of violaxanthin, zeaxanthin and β-carotene and a highly significant increase of lutein (more than 10-fold) compared to untreated cells, thus showing the ability of DIMEB to enhance carotenoid production (Figure 1A). After a seven-day treatment the intracellular levels of carotenoids resulted similar compared to the untreated cell cultures (Figure 1C). Intracellular quinone levels in treated and untreated *A. annua* suspension cultures were also measured. In comparison with the untreated cells, after three days of 50 mM DIMEB treatment, significant increases of intracellular levels of Q9 (about 3-fold) and Q10 (2.5-fold) were observed (Figure 2A). After seven days the intracellular Q9 level decreased by 20% compared to the content of the control cells while the Q10 level was very similar to the level of the untreated cells (Figure 2C). These results clearly indicate that DIMEB were able to induce an early increase of intracellular carotenoid and quinone content, which could be observed to a higher extent for lutein, Q9 and Q10. It has been shown that DIMEB can act as enhancer of secondary metabolite biosynthesis in different plant cell cultures alone or in combination with methyl jasmonate [5,6,12,13]. This activity was explained by the similarity of CD chemical structure to pectic oligosaccharides released by the cell wall after a fungal infection Thus, the DIMEB induction of carotenoid and quinone metabolism here detected, to our knowledge for the first time, could resemble a defense response of stressed plant cell cultures.

Since *A. annua* cell cultures had already been shown to produce extracellular amounts of the terpenoid artemisinin [11], the culture medium of 50 mM DIMEB treated cultures was analyzed for carotenoid and quinone contents in comparison with the medium of untreated cultures. The results showed a considerable increase of extracellular levels induced by the DIMEB treatment that could be detected for most carotenoids after three and seven days, while hardly detectable levels of carotenoids with the exception of lutein, were observed in the medium samples of untreated cultures. Moreover, in the medium of DIMEB treated cells a time-related increase of carotenoid levels was observed by analyzing three- and seven-day-treated samples (Figure 1B,D). When the medium of *A. annua* cell cultures was analyzed for quinones, a significant Q9 increase in the medium of three-day-treated cells was observed compared to the untreated samples, while no detectable Q10 levels were observed both in the medium of untreated and treated samples (Figure 2B). After 7 days, extracellular Q9 and Q10 levels significantly increased in the medium of treated cells compared to the control (Figure 2D).

These results indicate that the addition of DIMEB to *A. annua* suspension cultures not only induced an early increase of intracellular carotenoid and quinone content, but also an enhanced release of carotenoids and quinones into the culture medium. It is known that CDs are able to form inclusion complexes with non-polar compounds, favoring their accumulation in aqueous solution. In recent years, the complexation of hydrophobic antioxidant compounds, including carotenoids and quinones, in aqueous medium with molecules such as CDs has been reported in order to increase their stability, bioavalaibility and solubility (for a review [16]). Therefore this ability to form inclusion complexes with hydrophobic molecules, such as isoprenoids, could explain the increase of carotenoids and quinones released into the culture medium by *A. annua* cell cultures treated with 50 mM DIMEB, possibly as an effect of enhanced membrane permeability [17] or improved stability of the isoprenoid molecules in the medium. The addition of β-CDs to plant cell cultures to improve the production and the extracellular accumulation of various secondary metabolites such as resveratrol and phytosterols has been described in suspension cell cultures of *Vitis vinifera* and *Daucus carota*, respectively [5,6,7,8,9,10,11,12]. In a previous work, we showed the ability of β-CDs to improve the release of the sesquiterpene lactone artemisinin from *Artemisia annua* cell cultures [6]. The results of this work suggest that β-CDs could also be successfully used for enhancing the production and release of carotenoids and quinones in *A. annua* suspension cell cultures. Although plant *in vitro* cultures have received a lot of attention as an effective technology for the production of valuable secondary metabolites, however, in most cases, the desired products are stored, often as low amounts, within the cells and this prevents an efficient and continuous production of useful metabolites increasing the complexity and the cost of the whole process [1]. Finding out strategies to overcome these problems, by enhancing product yields and inducing the release or exudation of useful products into the culture medium can be actually helpful. The secretion of isoprenoids induced by DIMEB in the culture medium of *A. annua* cell cultures could be promising in view of making easier the isolation of these molecules to be exploited for the production of nutraceuticals, food additives or ingredients for cosmetics.

### 2.2. Expression of Isoprenoid Biosynthetic Genes and Isoprenoid Production

**Figure 3:**
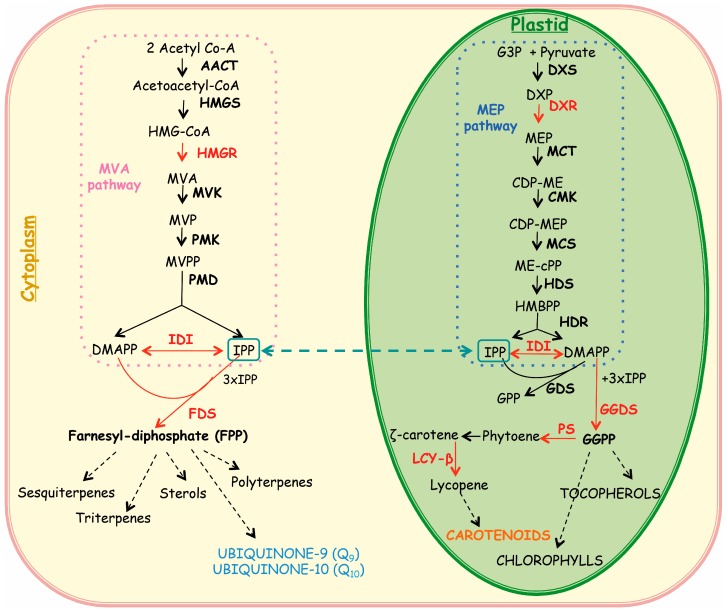
Isoprenoid biosynthetic pathways in the plant cell. Abbreviations used in **Cytosol**: HMG-CoA, Hydroxymethylglutaryl-coenzyme A; MVA, mevalonic acid; MVP, 5-phosphomevalonate; MVPP, 5-diphosphomevalonate; DMAPP, dimethylallyl diphosphate; IPP, isopenthenyl diphosphate; FPP, farnesyl diphosphate; AACT, acetoacetyl-coenzyme A thiolase; HMGS, 3-hydroxy-3-methyl-glutaryl coenzyme A synthase; *HMGR*, 3-hydroxy-3-methyl-glutaryl coenzyme A reductase; MVK, mevalonate kinase; PMK, phosphomevalonate kinase; PMD, diphosphomevalonate decarboxylase; *IDI*, isopentenyl diphosphate isomerase; *FDS*, farnesyl diphosphate synthase. Abbreviations used in **Plastid**: G3P, d-glyceraldehyde-3-phosphate; DXP, 1-deoxy-d-xylulose-5-phosphate; MEP, 2-*C*-methyl-d-erythritol-4-phosphate; CDP-ME, 4-(cytidine 5'-diphospho)-2-*C*-methyl-d-erythritol; CDP-MEP, 2-phospho-4-(cytidine 5'-dipospho)-2-*C*-methyl-d-erythritol; ME-cPP, 2-*C*-methyl-d-erythritol-2,4-cyclodiposphate; HBMPP, hydroxymethylbutenyl-4-diphosphate; GPP, geranyl diphosphate; GGPP, geranyl geranyl diphosphate; DXS, 1-deoxy-d-xylulose-5-phosphate synthase; *DXR*, 1-deoxy-dxylulose-5-phosphate reductoisomerase; MCT, 2-*C*-methyl-d-erythritol-4-(cytidyl-5-diphosphate) transferase; CMK, 4-cytidine 5'-diphospho-2-*C*-methyl-d-erythritol kinase; MCS, 2-*C*-methyl-d-erythritol-2,4-cyclodiphosphate synthase; HDS, hydroxy-2-methyl-2-(*E*)-butenyl 4-diphosphate synthase; HDR, hydroxy-2-methyl-2-(*E*)-butenyl 4-diphosphate reductase; GDS, geranyl diphosphate synthase; *GGDS*, geranylgeranyl diphosphate synthase; PS, phytoene syntase; LCY-B, lycopene-β-cyclase.

On the basis of the chemical similarity of β-CDs to pectic oligosaccharides released by the cell wall after a fungal infection, the early induction response after three days of treatment, mainly regarding lutein, Q9 and Q10, thus determining a total increase of intracellular content of isoprenoids in cell cultures of *A.* *annua* (Figure 1 and Figure 2), could be explained by the occurrence of a stress response. In *Vitis vinifera* β-CDs have been reported to act as true elicitors by activating different families of transcription factors regulating the expression of genes involved in the trans-resveratrol biosynthetic pathway [4,5,18]. In order to analyze the relationship between the transcript profile of biosynthetic genes and the production of isoprenoid compounds, the expression levels of 1-deoxy-d-xylulose-5-phosphate reductoisomerase (*DXR*), farnesyl diphosphate synthase (*FDS*), geranylgeranyl diphosphate synthase (*GGDS*), isopentenyl diphosphate isomerase (*IDI*), lycopene beta-cyclase **(***LYC-B*), phytoene synthase (*PS*) and 3-hydroxy-3-methyl-glutaryl-Coenzyme A reductase (*HMGR*) genes were determined in *A. annua* cell cultures treated for one and three days with DIMEB in comparison with untreated samples. These genes were chosen as key enzymes in the terpenoid biosynthetic routes, the cytosolic MVA and the plastidial MVA independent, as shown in Figure 3.

**Figure 4:**
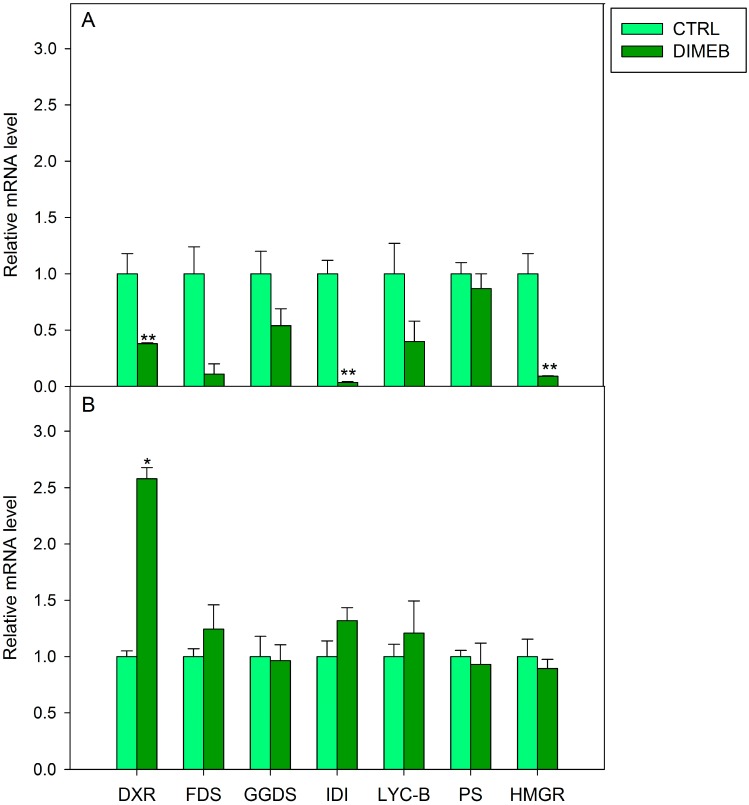
Relative mRNA level in *A. annua* cell cultures untreated (CTRL) or treated (DIMEB) with 50 mM DIMEB for one (**A**) and three (**B**) days. Data are expressed as mean ± standard deviation from three independent experiments, * *p* < 0.05; ** *p* < 0.01 in comparison with control assessed by Anova-one-way *post hoc* Holm-Sidak test.

The results of Real-Time PCR revealed that in *A. annua* cell cultures treated for one day with DIMEB, transcript accumulation of all the analyzed genes was lower than in untreated samples (Figure 4A), while in cells treated for three days the expression of *DXR* was significantly higher (more than 2.5-fold) compared to untreated samples. Slight increases were observed in the expression of *FDS*, *IDI* and *LYC-B* genes and no significant changes were detected for the other genes (Figure 4B). *DXR* encodes for a key enzyme in the plastidial MVA-independent pathway, catalyzing the biosynthesis of methylerythritol 4-phosphate (MEP), which is the first committed intermediate of the pathway [19]. *DXR* overexpressing *Arabidopsis thaliana* plants were reported to have an increased accumulation of plastid isoprenoids including chlorophylls and carotenoids [20]. Thus the up-regulation of *DXR* gene only observed after three days of DIMEB treatment could account for the increase of carotenoids in our cell system. The considerable increase of chlorophyll levels measured in DIMEB treated cells compared to untreated samples (data not shown) strengthened this hypothesis. On the other hand, a cross talk between the two isoprenoid routes has been demonstrated and an export of isoprenoid C5 units from the plastid to the cytosol can occur in higher plants [21] thus the enhancement of the plastidial route could influence also the formation of cytosolic isoprenoids such as quinones.

## 3. Experimental Section

### 3.1. A. annua Suspension Cultured Cells

*A. annua* suspension cultured cells (SCC) were established and maintained as described by Caretto *et al.* [11] in G6 medium. Briefly, suspension cultures were maintained in MS medium [22] supplemented with 2 mg/L of 2,4-dichlorophenoxyacetic acid (2,4-D) and 0.15 mg/L 6-benzylaminopurine (BAP). Cultures were incubated on a rotary shaker (120 rpm) at 25 °C under continuous fluorescent white light (125 µmol·photons/m^2^·s) and were subcultivated every 35 days in 500 mL Erlenmeyer flasks by transferring 15 mL of the 35-day-old suspensions into 85 mL fresh G6 medium. Growth of SCC was monitored by measuring dry weight (DW) increase during the culture cycle. Cell viability was assayed using the fluorescein diacetate staining method [14].

### 3.2. Elicitation of A. annua SCC

Elicitation experiments were performed in triplicate using exponentially growing (15-day-old) *A. annua* SCC [11]. SCC were centrifuged at 300× *g* for 10 min, and medium was discarded. Cells (2.5 g fresh weight) were transferred to a 100 mL Erlenmeyer flask containing 10 mL fresh G6 liquid medium (control) or 10 mL G6 medium supplemented with 50 mM DIMEB (Sigma, St. Louis, MO, USA). SCC were incubated for 3 and 7 days as suggested by previous results [6]. After elicitation, cells were separated from the culture medium under gentle vacuum, using Miracloth filters (Calbiochem, Los Angeles, CA, USA) then washed three times (5 min each) with 150 mL total fresh G6 medium. Filtered cells were frozen and lyophilized overnight (Labconco, Kansas City, MO, USA) and both, cells (50 mg) and the spent medium (3 mL) were used for subsequent analysis.

### 3.3. Carotenoid and Quinone Extraction from A. annua Cells and Spent Medium

Carotenoid and quinone contents were determined according to the method of Sadler *et al.* [23] and Perkins-Veazie *et al.* [24] with some modification. Lyophilized cell samples (50 mg) and the spent medium (3 mL) were extracted with 5 mL (0.05% *w*/*v*) of butylated hydroxytoluene (BHT) in acetone, 5 mL 95% ethanol and 10 mL hexane for 15 min under stirring at 180 rpm and then for 5 min after the addition of distilled water (3 mL). The extracts were centrifuged at 4000× *g* for 10 min, and extracts were collected and dried under vacuum. The extraction from the spent medium was carried out as described for cells.

### 3.4. HPLC Analysis of Carotenoid and Quinone

**Figure 5:**
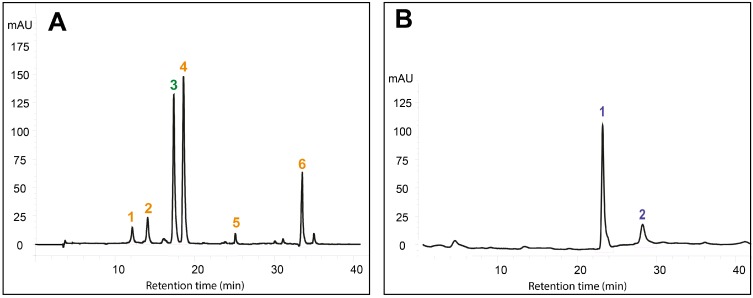
HPLC analysis of *Artemisia annua* suspension cell extracts recorded at 475 nm (**A**) and at 275 nm (**B**). Cells were treated with 50 mM DIMEB for three days. Peaks in (**A**) are (1) violaxanthin; (2) neoxanthin; (3) chlorophyll b; (4) lutein; (5) zeaxanthin; (6) β-carotene. Peaks in (**B**) are (1) Q9; (2) Q10.

Samples redissolved in hexane were dried at speedvac, redissolved in ethyl acetate (100 µL for cells and 50 µL for medium samples) and immediately analyzed by HPLC. Quali-quantitative analyses were carried out using an Agilent 1100 Series HPLC system (Agilent Tecnologies, Waldbronn, Germany) as described by Fraser *et al.* [25] but slightly modified. Carotenoid and quinones were separated using a reverse-phase C_30_ column (5 µm, 250 × 4.6 mm) (YMC Inc., Wilmington, NC, USA) with mobile phases consisting of methanol (A), 0.2% ammonium acetate aqueous solution/methanol (20/80 *v*/*v*) (B), and *tert*-methyl butyl ether (C). The elution was as follows: 0 min, 95% A and 5% B; 0 to 12 min, 80% A, 5% B, and 15% C; 12 to 42 min, 30% A, 5% B, and 65% C; 42 to 60 min, 30% A, 5% B, and 65% C; 60 to 62 min, 95% A, and 5% B. The column was re-equilibrated for 10 min between runs. The flow rate was 1.0 mL/min, and the column temperature was maintained at 25 °C. The injection volume was 10 L. Absorbance was registered by diode array at wavelengths of 475 nm for carotenoids and 275 nm for quinones. Carotenoids and quinones were identified by comparing their retention times and UV–vis spectra to authentic standards. An example of chromatographic profile of carotenoids and quinones in *A. annua* cell cultures was reported in Figure 5. Carotenoid standards were purchased from Sigma–Aldrich (Milan, Italy), Cayman Chemicals (Ann Arbor, MI, USA), and Extrasynthese (Genay CEDEX, France).

### 3.5. Relative Expression Analysis of Genes of Isoprenoid Biosynthesis

*A. annua SCC* were filtered, frozen in liquid nitrogen, lyophilized and reduced to a powder. Total RNA was isolated from lyophilized cell samples (5 mg) by using the “SV Total RNA Isolation System” (Promega, Milano, Italy), following the manufacturer’s instructions. The RNA quality was checked by agarose gel electrophoresis and quantified using the Infinite^®^ M200–Pro spectrofluorometer (TECAN, Männedorf, Switzerland), using Tecan plate NanoQuant. Four micrograms of total RNA was treated with RQ1 RNase-Free DNase (Promega, Milano, Italy) and the cDNAs were synthesized using random primers and the GoScript™ Reverse Transcription System (Promega, Fitchburg, MA, USA), according to the manufacturer’s instructions. Reverse Transcription-PCR cycles were as follows: 25 °C for 5 min, 42 °C for 1 h and 70 °C for 15 min.

### 3.6. Real-Time PCR Experiment

Quantitative real-time PCR experiments were performed in an Applied Biosystems 7500 apparatus using the sequence-specific primer set (900 nM each), specific probes (200 nM each) (Table 1) labelled at the 5'-end with 6-carboxy-fluorescein (FAM) and at the 3'-end with tetramethylrhodamine (TAMRA), 0.5 µL of the first strand cDNA and 12.5 µL of 2X TaqMan Universal PCR Master Mix (Applied Biosystems, Foster City, CA, USA) in a total volume of 25 µL. Negative controls were performed by omitting the template in the PCR reaction mix. Experiments were conducted in triplicate and using cDNAs obtained from at least two independent experiments. Real-time PCR cycles were as follows: 50 °C for 2 min (1 hold); 95 °C for 10 min (1 hold); 95 °C for 15 s, 60 °C for 1 min (40 cycles). Quantification of transcripts was carried out using the comparative quantitation module, as described for the ABI 7500 Sequence Detection System, (User Bulletin 2; Applied Biosystems) based on the 2^−ΔΔ*C*t^ method [26]. The relative expression was normalized against β-actin and calculated using the untreated samples as a calibrator, whose expression was arbitrarily set to one.

**Table 1:** Primers and probe sequences used for quantitative Real-Time PCR analysis in *A. annua* cell cultures.

Gene		Sequence	Accession Number
*HMGR*	Forward Reverse Probe	5'-CATGCTTGAACCTACTTGGAGTCA-3' 3'-CAACACCGAACCAGCAACTATC-5' 5'-TGCGTGCATAGAATCACCAGGCTCA-3'	AF142473.1
*DXR*	Forward Reverse Probe	5'-CCCGTCTTGATCTTTGCAAGTT-3' 3'-GCAGAACAGCCAAATGCATT-5' 5'-AAGCACCGGACAACGTGAAATACCCG-3'	AF182287.2
*IDI* *	Forward Reverse Probe	5'-CAGACTTAGGTGAGGAGGGTCTCA-3' 3'-CCCACCACTTGAACAAGAAATTG-5' 5'-CTGTCGCCGTGGTTCAGGATTGTTG-3'	DQ666334.1
*FDS*	Forward Reverse Probe	5'-TCACCGCCGAATTGTTCA-3' 3'-TGCTTGTCAAGATCCTCTCCAA-5' 5'-ACTCATTTTACCTTCCAGTTGCCTGTGCAC-3'	U36376.1 AF112881.1
*GGDS*	Forward Reverse Probe	5'-AGGTTGTTTGCTGAGGAGTTGTT-3' 3'-CCACACCCCTCTCTGATTCAA-5' 5'-CCGAGGCGAAGCAGCAGTTGG-3'	EY076993.1 EY082045.1
*PS* *	Forward Reverse Probe	5'-GGGCGTATGTAAGCAAACCAA-3' 3'-CTTGATGATGCTGGTACAAGTGATT-5' 5'-AAGATAGTTGCTTTGCCTCTGGCATATGCA-3'	EY033375.1
*LCYB* *	Forward Reverse Probe	5'-TTTAGAAGGCACAAGACGGTTTT-3' 3'-AAAGTGAATAACTCAGGCAGAAACAA-5' 5'-ACCTCGTTACTGGCATGGGTTCTTGTCATC-3'	EY092112.1
β-action	Forward Reverse Probe	5'-CCATTGGTGCTGAGAGGTTCA-3' 3'-GCAGCTTCCATTCCGATCA-5' 5'-TGCCCTGAGGTCTTGTTCCAACCTTC-3'	EU531837.1

* *A. annua* *IDI*, (DQ666334.1) PS (EY033375.1) and *LYC-B* (EY092112.1) sequences were identified by searching in the *A. annua* EST sequences database on the basis of *Helianthus annuus* homologous genes.

### 3.7. Statistical Analysis

Results are presented as the mean value ± standard deviation (SD) of three independent experiments. Data were analyzed statistically by Anova-one-way *post hoc* Holm-Sidak test, using SigmaStat software Version 3.1 (SPSS Inc., Chicago, IL, USA). Significance level was set at 5%, 1% and 0.1%.

## 4. Conclusions

Suspension cell cultures of *Artemisia annua* have revealed to be a valuable system for evaluating the ability of DIMEB to enhance the intracellular and extracellular production of important isoprenoid molecules, such as carotenoids and quinones. In particular, the application of 50 mM DIMEB for three days was useful to induce a considerable significant increase of intracellular lutein, Q9 and Q10 contents compared to untreated cell cultures. In addition, the release of carotenoids and quinones into the culture medium of DIMEB-treated cell cultures was significantly enhanced in comparison with control cultures. Real Time PCR analysis revealed an up-regulation of *DXR* gene thus suggesting that the DIMEB induced increase of carotenoids and quinones could be due to the elicitation of the plastidial isoprenoid biosynthetic route.
